# CARF and WYL domains: ligand-binding regulators of prokaryotic defense systems

**DOI:** 10.3389/fgene.2014.00102

**Published:** 2014-04-30

**Authors:** Kira S. Makarova, Vivek Anantharaman, Nick V. Grishin, Eugene V. Koonin, L. Aravind

**Affiliations:** ^1^National Library of Medicine, National Center for Biotechnology Information, National Institutes of HealthBethesda, MD, USA; ^2^Departments of Biophysics and Biochemistry, Howard Hughes Medical Institute, University of Texas Southwestern Medical CenterDallas, TX, USA

**Keywords:** CRISPR, Rossmann fold, beta barrel, DNA-binding proteins, phage defense

## Abstract

CRISPR-Cas adaptive immunity systems of bacteria and archaea insert fragments of virus or plasmid DNA as spacer sequences into CRISPR repeat loci. Processed transcripts encompassing these spacers guide the cleavage of the cognate foreign DNA or RNA. Most CRISPR-Cas loci, in addition to recognized *cas* genes, also include genes that are not directly implicated in spacer acquisition, CRISPR transcript processing or interference. Here we comprehensively analyze sequences, structures and genomic neighborhoods of one of the most widespread groups of such genes that encode proteins containing a predicted nucleotide-binding domain with a Rossmann-like fold, which we denote CARF (CRISPR-associated Rossmann fold). Several CARF protein structures have been determined but functional characterization of these proteins is lacking. The CARF domain is most frequently combined with a C-terminal winged helix-turn-helix DNA-binding domain and “effector” domains most of which are predicted to possess DNase or RNase activity. Divergent CARF domains are also found in RtcR proteins, sigma-54 dependent regulators of the *rtc* RNA repair operon. CARF genes frequently co-occur with those coding for proteins containing the WYL domain with the Sm-like SH3 β-barrel fold, which is also predicted to bind ligands. CRISPR-Cas and possibly other defense systems are predicted to be transcriptionally regulated by multiple ligand-binding proteins containing WYL and CARF domains which sense modified nucleotides and nucleotide derivatives generated during virus infection. We hypothesize that CARF domains also transmit the signal from the bound ligand to the fused effector domains which attack either alien or self nucleic acids, resulting, respectively, in immunity complementing the CRISPR-Cas action or in dormancy/programmed cell death.

## Introduction

In prokaryotes CRISPR-Cas systems (Clustered Regularly Interspaced Short Palindromic Repeats- CRISPR-associated genes) code for RNA-dependent self–non-self recognition mechanisms, which are partially analogous eukaryotic RNA interference (RNAi) systems, and serve as an adaptive immunity system against invasive nucleic acids. The CRISPR-Cas system incorporates fragments of virus or plasmid DNA into the CRISPR repeat cassettes and employs the processed transcripts of these spacers as guide RNAs to cleave the cognate foreign DNA or RNA. Recently, the type-II CRISPR systems have been used as biotechnological reagents of targeted mutagenesis, genome editing or gene-inactivation in eukaryotes (Jinek et al., 2013; Mali et al., 2013; Niu et al., 2014). Many CRISPR-Cas systems are associated with genes that appear not to be directly implicated in spacer acquisition, CRISPR transcript processing or the restriction of the invasive nucleic acids known as interference (Makarova et al., 2011a,b; Wiedenheft et al., 2012; Koonin and Makarova, 2013). The most common among such genes (the csm6/csx1-like genes) encode experimentally uncharacterized or poorly characterized proteins that belong to COG1517 (Makarova et al., 2006, 2011b). Structures of four proteins from this family have been experimentally determined and it has been shown that they all share a distinct Rossmann-fold-like domain that we here denote CARF (CRISPR-Cas Associated Rossmann Fold). In addition, most of the CARF domain proteins contain a winged HTH (wHTH) DNA-binding domain immediately C-terminal of CARF (Lintner et al., 2010; Kim et al., 2013). It has been hypothesized that these proteins are CRISPR-Cas system-specific, allosterically controlled transcriptional regulators, with the Rossmann-like domain binding an unknown nucleotide (Lintner et al., 2010). Recently, involvement of the Csx1 protein in the interference associated with type III-B CRISPR-Cas systems in *Sulfolobus islandicus* has been demonstrated (Deng et al., 2013). Furthermore, deletion of the *csm6* gene results in disruption of CRISPR-based immunity in *Staphylococcus epidermidis* (Hatoum-Aslan et al., 2013).

Despite the progress in the structure analysis and the availability of first experimental clues, the specific biochemical roles of the CARF proteins in the CRISPR-Cas systems and beyond remain largely obscure. Many CARF-domain proteins possess additional C-terminal domains that include both DNases, in particular those of the Restriction Endonuclease (REase) fold (Makarova et al., 2006), and RNases, such as members of the RelE (Koonin and Makarova, 2013) and HEPN families (Anantharaman et al., 2013). This observation led to a hypothesis that these proteins can be involved in immunity mechanisms complement the activity of the core CRISPR-Cas systems by targeting self or invasive nucleic acids (Makarova et al., 2012, 2013; Anantharaman et al., 2013). Action against self nucleic acids could augment the immunity of a population of prokaryotic cells in two ways: first, by inducing dormancy and thus “buying time” for the immune system to spring into action, or second, by inducing programmed cell death of the host when CRISPR-Cas fails to stop virus propagation (Makarova et al., 2012, 2013; Koonin and Makarova, 2013). Here we present an in-depth comparative genomic and phylogenetic analysis of the CARF (COG1517) superfamily in an attempt to shed more light on the function and evolution of these proteins.

## Results

### Sequence analysis and identification of new members of the CARF superfamily

**Figure 1:**
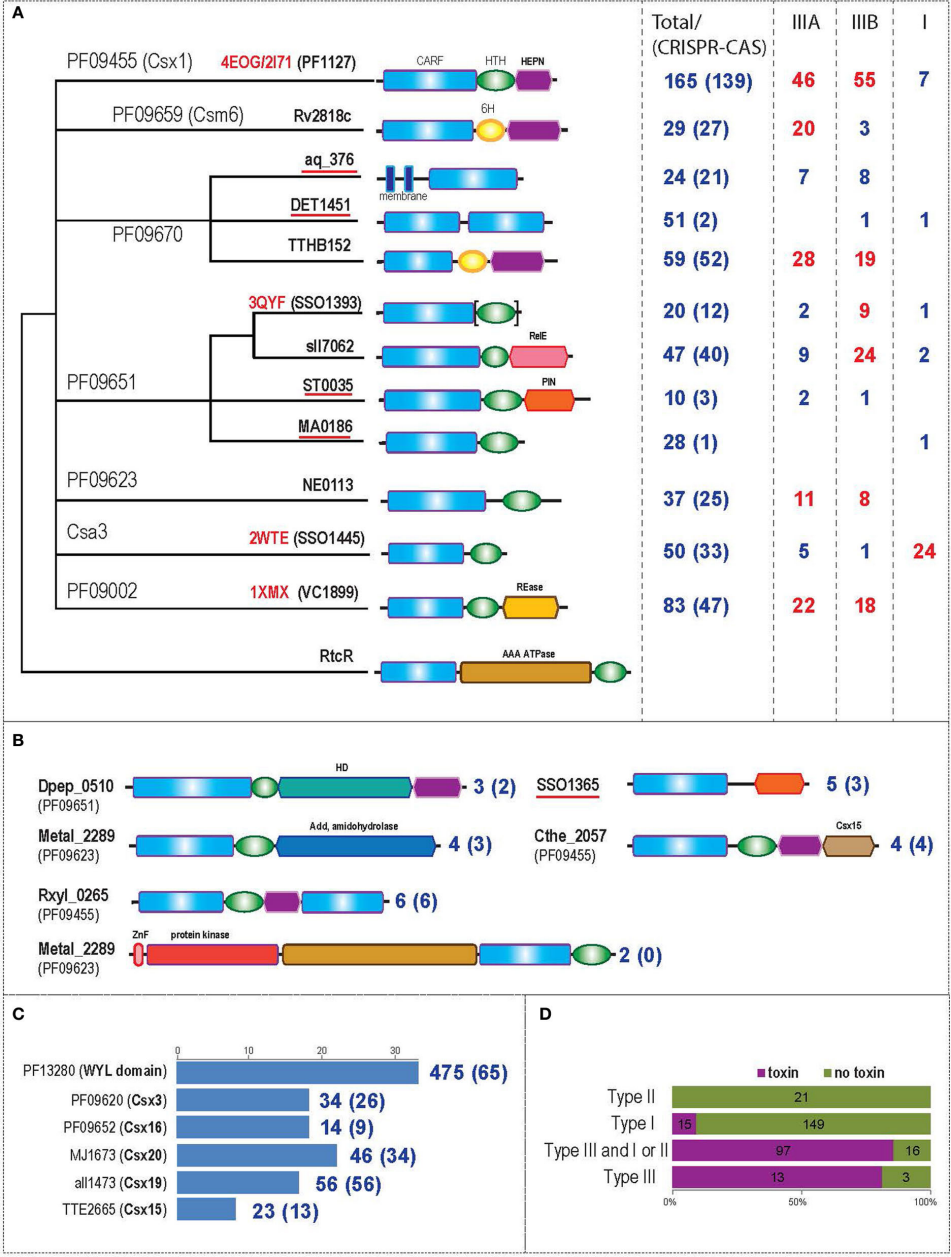
**Comparative genomic analysis of CARF domain-containing proteins. (A)** scheme of the relationships between major CARF families, their domain architectures and association with CRISPR-Cas system types. The dendrogram shows the relationship between CARF domain containing families. The clustering is based on sequence and structure similarity analysis as described under Materials and Methods; unresolved relationships are shown as a multifurcation. The pfam ID or other recognized family description is provided for each of the seven major groups. A typical member of a family (either locus tag of a representative protein or a pdb identifier) is shown for each terminal node; subfamilies that have not been described previously are underlined. The typical domain architecture is shown for each family. The domain name is shown above the corresponding shape the first time it appears. Brackets indicate that in several proteins in the respective family the domain is missing. In the first column on the right hand side, the number of proteins in the respective family is indicated, and the number of proteins encoded in the vicinity of *cas* genes is shown in parentheses. In the second, third and fourth columns, the number of genes of each family that are specifically associated with CRISPR-Cas systems of types III-A, III-B, and I are shown (the numbers representing a substantial fraction of the family are highlighted in red). **(B)** Domain organization of several minor CARF domain-containing families. Designations are as in Figure 1A. **(C)** Protein families associated with genes encoding CARF domains. The histogram shows how many times each family was identified in the vicinity of CARF domain-containing genes; the scale is shown above the histogram. Only the most frequently co-occurring families outside the set of recognized *cas* genes are shown. The numbers on the right hand side reflect the results of a reverse analysis when neighborhoods of the genes from each family were analyzed for the presence of *cas* genes. The total number of genes and the number of genes in the vicinity of known *cas* genes (in parentheses) are indicated. **(D)** Association of CARF domains with (predicted) toxin domains in the three types of CRISPR-Cas systems. The histogram shows the co-occurrence of CARF proteins with toxin domains separately for the three CRISPR-Cas system types; the type III systems are additionally partitioned into those that co-occur with type I or type II in the same genome and those that represent the sole instance of CRISPR-Cas in the respective genomes.

We used several approaches to identify CARF superfamily proteins. First, a CDD search was employed to identify all proteins in 2262 complete genomes (as of February 2013) that could be assigned to previously identified CARF families [namely COG1517, PF09455, PF09670, PF09659, PF09651, PF09623, PF09002, Csa3 (Lintner et al., 2010; Makarova et al., 2011b)]. Representatives of each family were used as queries for PSI-BLAST using the search strategy described in the Materials and Methods section (Altschul et al., 1997). Putative new members were validated using HHpred search (Soding et al., 2005). The same methods were used to identify other domains fused to CARF domains (Supplementary File 1). For further analysis incomplete protein sequences were discarded. The final data set included 1441 proteins (Supplementary File 1). This set was further clustered to generate a non-redundant subset (635 proteins) using BLASTCLUST (Wheeler and Bhagwat, 2007) with a length coverage cutoff of 0.8 and a score coverage threshold (bit score divided by alignment length) of 0.8. For this representative subset of 635 CARF domain-containing proteins, analysis of domain architecture and gene neighborhoods was performed as described under Materials and Methods. Because the extensive sequence divergence of the CARF domains results in saturation of substitutions and prevents building a high quality alignment for phylogenetic analysis, the relationships between families were determined approximately, on the basis of their similarity in HHpred searches (Figure 1 and Supplementary File 2).

Figure 1 shows the relationships between the CARF families, their domain organization and association (if any) with different types of CRISPR-Cas systems. The results of this analysis suggest that the CARF superfamily could be classified into at least 12 distinct major families with 10 or more representatives each and several minor families (Figures 1A,B, Supplementary File 1). In addition to the aforementioned CARF domain families, HHpred search using pfam09659 as the query identified significant sequence similarity between the CARF domain and an uncharacterized N-terminal domain of RtcR (Supplementary File 2), which is the regulator of the *Rtc* RNA repair system that consists of the 3′-terminal phosphate cyclase RtcA, and RNA ligase RtcB (Genschik et al., 1998; Chakravarty et al., 2012). Although this domain occurs in distinct protein architectural and genomic contexts (see below), it shares distinct sequence motifs with the CARF domains to the exclusion of other Rossmann fold domains. Hence we consider the predicted nucleotide-binding domain of RtcR a divergent member of the CARF superfamily.

### Structural features of CARF domain proteins

**Figure 2:**
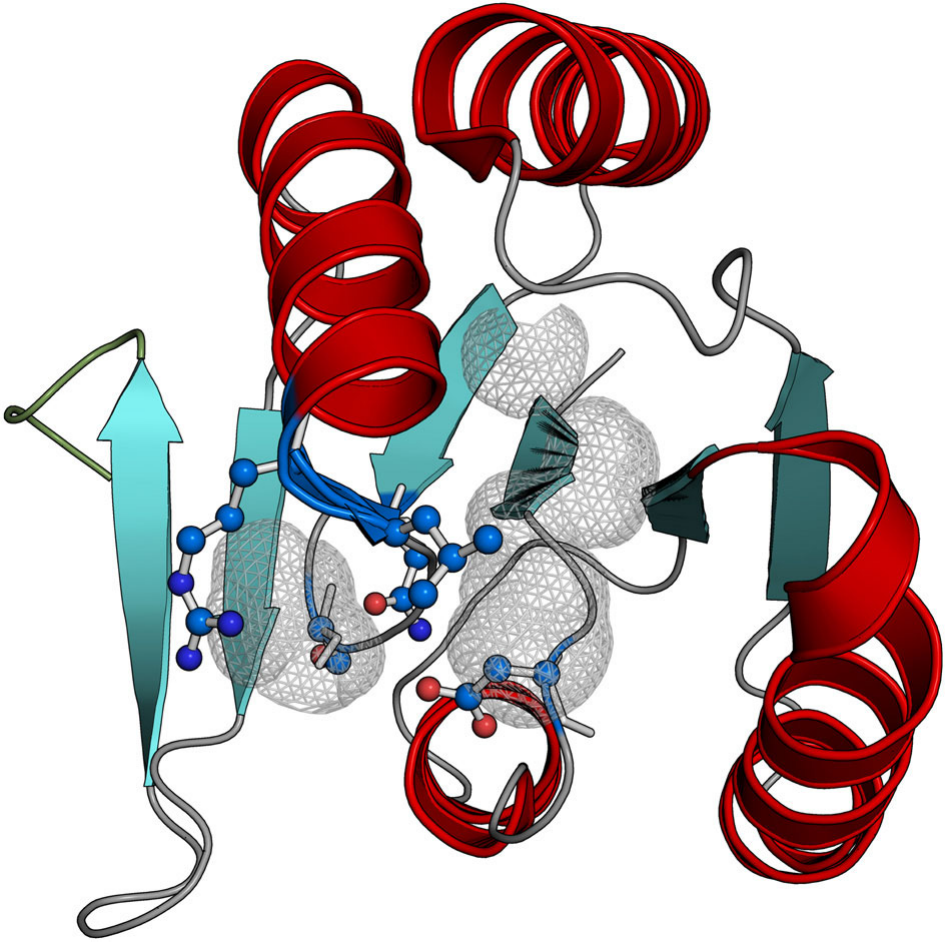
**Structure of the VC1899 CARF domain**. This version of the CARF domain contains no elaborations or inserts observed in certain other CARF domains. The predicted active site pocket was identified using probe of 2 solvent or greater radii (gray mesh) and the predicted ligand-interacting residues the pocket are also shown.

The availability of five crystal structures of CARF domain proteins along with the above sequence analysis provides for a more detailed understanding of the conserved structural features of the superfamily and their functional implications. The core of the CARF domain is a six-stranded Rossmann-like fold with the core strand-5 and strand-6 forming a β-hairpin (Figure 2). The main regions of sequence conservation are associated with strand-1 and strand-4 of the core domain: the end of strand-1 is often characterized by a polar residue, typically with an alcoholic side chain (S/T), whereas immediately downstream of strand-4 is a highly conserved basic residue (K/R) often associated with [DN]X[ST]XXX[RK] signature. The position of these characteristic motifs is typical of the location of substrate-binding sites across a diverse range of Rossmann-like domains (Anantharaman and Aravind, 2006; Burroughs et al., 2006, 2009) with the implication that the ligand-binding capability is conserved throughout the CARF superfamily. Consistent with this prediction, probing the active site with a probe of 2 or more solvent radii shows the presence of a conserved pocket that is formed largely by the residues from the aforementioned motifs associated with strand-1 and strand-4 (Figure 2, Supplementary File 3). The conservation of K/R after strand-4 and its location in the pocket is consistent with the proposal of a nucleotide or nucleotide-derived molecule being the primary ligand of the CARF domains (Lintner et al., 2010). However, the RV2818 and RtcR families mostly lack the positively charged residue downstream of strand-4 suggesting that they might bind distinct ligands.

**Figure 3:**
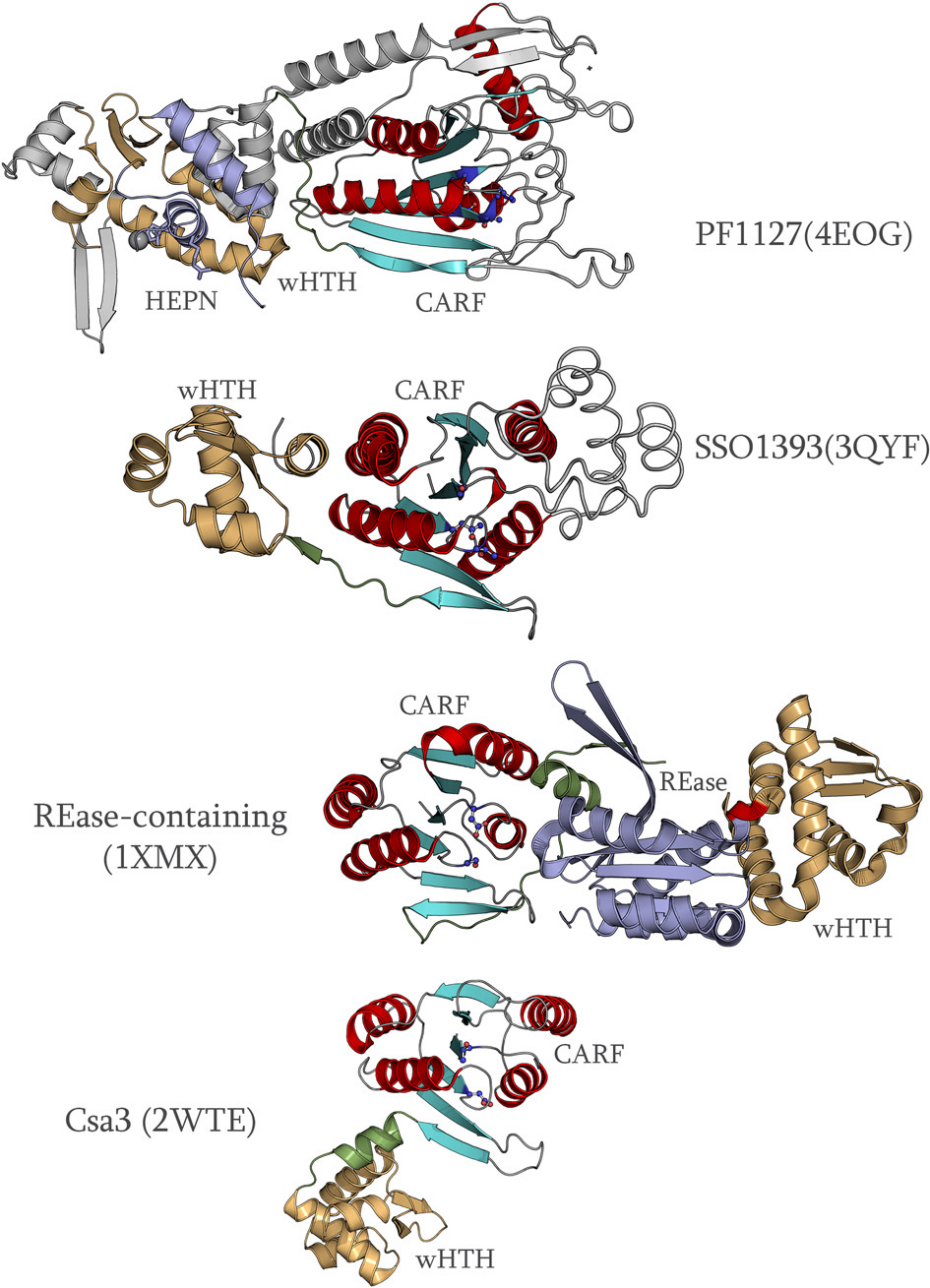
**Comparison of the structures of multiple CARF proteins**. The CARF domains of all proteins were aligned and then separated for clarity. The different spatial orientations of the C-terminal domains are shown with respect to the CARF domain. The linker between the CARF domain and the C-terminal domains is colored green, the wHTH or the equivalent domain is rendered in white, and the C-terminal effector domain is colored purple. Inserts within the CARF domain are colored gray and are shown in “wire” representation. A domain of uncertain origin in PF1127 is colored gray and is shown as ribbon.

Examination of the structures also shows that the core fold of the CARF domain is prone to considerable divergence due to several distinct inserts (Figure 3). For example, in the group that consists of the SSO1393, sll7062, ST0035, and MA0186 families, there is an α-helical bundle inserted immediately after strand-1. Likewise, in the PF1127 family, a β-hairpin is inserted after strand-1 and multiple additional inserts are present after strand-2, strand-3 and in the β-hairpin formed by strand-5 and strand-6 (Figure 3). Based on the sequence alignments, we also detected smaller but comparable inserts after strand-1 in most members of the Aq_376 group and several members of the DET1451 group. These inserts typically are packed around the active site and form a “cap” that appears to shelter and augment the conserved ligand-binding site. The repeated emergence of inserts in similar locations in different families suggests that they might be determinants of ligand diversity across the CARF superfamily.

Another striking feature revealed by the comparison of the available structures is the diversity of spatial positions of the C-terminal wHTH and effector domains (Figure 3) vis-à-vis the CARF domain. This diversity of spatial positions is in sharp contrast to the strong positional polarity that is typical of prokaryotic one-component transcription factors with respect to their upstream ligand-binding domains (Aravind et al., 2010). Instead, it appears likely that the spatial organization of the C-terminal domains reflects optimization for transmitting the signal generated by the bound ligand to different C-terminal effector domains. This observation is compatible with the proposal that in most members of the CARF superfamily ligand binding is not directly linked to transcription but rather affects other DNA-associated activities (See discussion below).

### Domain architectures of CARF superfamily proteins

The majority of the families contain a wHTH domain downstream of the CARF domain (Figures 1A,B, Supplementary File 2). In the PF09659 and PF09670 related families, we were unable to identify a HTH domain; instead, proteins in both these families contain a distinct, conserved alpha-helical region (6H domain) (Supplementary File 3). In the largest family (PF09455), the wHTH domain cannot be identified by sequence similarity searches (Kim et al., 2013) but an α-helical domain of uncertain provenance, potentially derived from a wHTH is present at the C-terminus, and harbors a partly disordered insertion that contains a highly modified remnant of the HEPN domain. In addition to the previously described fusions to DNases and RNases, several new domain architectures were identified in this analysis, namely (1) fusion of two CARF domains, (2) a membrane-associated CARF, (3) fusion with a HD phosphoesterase domain, (4) fusion to a TIM barrel adenosine deaminase Ada domain the enzyme that catalyzes deamination of adenosine to inosine in the purine salvage pathway (Nygaard, 1977; Holm and Sander, 1997). Notably, fusion of the CARF domain with nuclease domains of the same family might have occurred independently on several occasions. In particular, we detected at least four distinct CARF families associated with the HEPN domain and two families associated with the PIN domain (Figures 1A,B). Overall, most of the C-terminal catalytic domains of the CARF superfamily proteins are predicted to be nucleases or other enzymes targeting nucleic acids (Makarova et al., 2013).

A small family consists of large multidomain proteins in which a Zn ribbon, a serine/threonine/tyrosine protein kinase and a distinctive AAA+ ATPase domain with an arginine finger within the P-loop motif are fused upstream of the CARF and wHTH domains (Figure 1B). In this case, the CARF domain might function as part of a signal transduction pathway mediated by the kinase. The RtcR proteins in addition to the divergent CARF domain contain a NtrC-like AAA+ ATPase and HTH domains. Furthermore, BLAST search initiated with the CARF-like domain of RtcR detects high similarity with a family of proteins that, similar to RtcR, contain NtrC-like AAA+ ATPase and HTH domains but are not linked to *Rtc* system. Instead these proteins are often associated with restriction-modification (R-M) systems (Supplementary File 4). One of the close homologs of these proteins, PspF, which contains AAA ATPase and HTH domains only, has been shown to be involved in sigma-54 dependent activation of membrane-associated phage shock protein (PSP) system in response to phage infection and other stress factors (Model et al., 1997; Joly et al., 2009, 2010). Thus, these systems are likely to function as sigma-54 dependent activators of their respective downstream genes, with the NtrC-like AAA+ domain binding the sigma factor. In these proteins, CARF domains might sense ligands generated during or after phage infection, such as RNA with 2′–3′ cyclic phosphate ends or a phage-specific nucleotide to regulate either RNA repair or DNA restriction. Thus, the central functional theme for the majority of CARF superfamily domains, whether associated with CRISPR-Cas systems or not, seems to be antivirus defense and stress response.

### The WYL domain and Cas protein families are enriched in gene neighborhoods of the CARF superfamily

To further characterize potential functional partners of the CARF proteins, we analyzed their genomic context by examining both known and new proteins families in the respective genomic neighborhoods. All gene products from these neighborhoods were collected, clustered using BLASTCLUST and analyzed using PSI-BLAST to further expand the respective families. The most common families associated with the CARF-domain proteins are shown in Figure 1C.

The WYL (named for three conserved amino acids found in a subset of domains of this superfamily) domain proteins are most abundant. Recently, it has been shown that a WYL domain protein (sll7009) is a negative regulator of the I-D CRISPR-Cas system in *Synechocystis* sp. (Hein et al., 2013). Further analysis of the WYL domain showed that the domain boundaries, as currently defined in the Pfam database (PF13280), are inaccurate because they encompass both a copy of the domain WYL domain (Supplementary File 5) and an additional C-terminal extension which is found primarily in the subset of WYL proteins with wHTH domains. HHpred searches revealed similarity of the refined WYL domain with SH3 β-barrel fold related to Sm domains (Supplementary File 2). Additionally, these searches showed that the uncharacterized Pfam DUF2693 family and the YolD family encoded in SOS DNA repair-associated operons (Permina et al., 2002; Aravind et al., 2013) are also members of the WYL domain superfamily (Supplementary File 2). Although the WYL domain was originally named for the 3 eponymous amino acids, examination of the refined and expanded alignment generated in the course of this work showed that these residues are not strongly conserved throughout the family. Rather, the conservation pattern includes four basic residues and a position often occupied by a cysteine (Supplementary File 5), which are predicted to line a ligand-binding groove typical of the Sm-like SH3 β-barrels (Gutierrez et al., 2007). Given that WYL domains often occurs in two copies in the same polypeptide or are encoded alongside other genes encoding multi-WYL proteins, it is conceivable that they form torroidal multimeric assemblies similar to other Sm-like SH3 β-barrels with a central ligand-binding channel (Schumacher et al., 2002).

In terms of domain architectures, WYL domains are most frequently associated with different predicted DNA-binding N-terminal wHTH domains. However, similar to the CARF domains, WYL domains also show fusions to several enzymatic domains (Supplementary File 6). In some of the type I CRISPR-Cas systems, a WYL domain is fused to the Cas3 protein which consist of a HD phosphoesterase domain and Superfamily-II helicase module. Additionally, WYL domains combine with 3′→5′ exoRNase, Mrr-like REase, HNH endonuclease, Superfamily-I helicase, AbiGII-like nucleotidyltransferase (DUF1814), BRCT, and TerB domains (Anantharaman et al., 2012). These fusions, the relationship between the WYL domain and the Sm-like domains, and the sequence conservation pattern of the WYL domain together seem to suggest that this is another ligand-sensing domain that could bind negatively charged ligands, such as nucleotides or nucleic acid fragments, to regulate CRISPR-Cas and other defense systems such as the abortive infection AbiG system (O'Connor et al., 1996; Makarova et al., 2013).

Several *cas* genes are enriched in the gene neighborhoods of the CARF superfamily (Figure 1C, Supplementary File 7). One of these, csx19, is always associated with CRISPR-Cas systems, and is predicted to represent a diverged version of the RAMP domain (RRM-like fold) that is found in many Cas proteins (Makarova et al., 2011a). Thus, colocalization of the csx19 genes with the genes encoding CARF domain proteins might simply reflect their shared association with the CRISPR-Cas systems rather than a direct functional link. In addition, *cas* genes of another, less common family, Csx15, are fused to the genes coding for CARF domain proteins on several occasions (Figure 1B). The Csx15 proteins show no significant similarity to any known domains, and their functions remain obscure. However, the presence of several highly conserved residues, namely two histidines, glutamate, and arginine are reminiscent of active site residues of metal-independent RNases (Zhang et al., 2012) and could be potentially involved in catalysis (Supplementary File 7). This together with the CARF domain fusions (Figure 1B), suggest that Csx15 might be a novel nuclease.

### Strong link between CARF-containing proteins and CRISPR-Cas systems

The association of CARF domain-containing proteins with CRISPR-Cas systems, especially those of type III, has been noted previously (Makarova et al., 2011a,b; Anantharaman et al., 2013; Koonin and Makarova, 2013). Here we sought to identify specific associations with CRISPR-Cas systems for each major family of CARF-domain proteins separately. The assessment was based on the proximity of the respective genes to CRISPR-Cas loci. Most of the 12 major CARF families are indeed typically found in vicinity of other *cas* genes (Figure 1A, Supplementary File 8), with the exception of DET1451, MA0186, and the divergent RtcR-like family. Those families of CARF-domain proteins that are associated with CRISPR-Cas systems most often are contained within type III CRISPR-Cas systems, and some show specific preference for type III-A or III-B. All these CARF domain protein families possess a third domain, a nuclease, which is predicted to function as a toxin that targets non-self or self-nucleic acids (Koonin and Makarova, 2013). The only CARF family (Csa3) that displays clear affinity to type I systems, and subtype I-A in particular, lacks a C-terminal catalytic effector domain. However, these associations notwithstanding, there are genes in each CARF family that are not linked to CRISPR-Cas and thus might not be functionally involved in the CRISPR-Cas-mediated defense. Some of the CARF genes that are not linked to CRISPR-Cas (e.g., Daci_4198 from *Delftia acidovorans*) of the VC1899 family (PF9002) are embedded within a novel Type-VII secretion system gene cluster predicted to function as a DNA-transfer agent and additionally encompassing multiple Ter genes that have been implicated in phage restriction (Anantharaman et al., 2012).

### CARF domain proteins containing a C-terminal effector domain belong to type III CRISPR-Cas systems

CARF domain-containing proteins are present in 145 genomes (among the representative set of 659 complete archaeal and bacterial genomes) of which only 9 genomes possess neither *cas1* nor *cas10* (the signature protein families of CRISPR-Cas systems), suggesting a strong link of these proteins to CRISPR-Cas (Supplementary File 9). Type III CRISPR-Cas systems often co-occur with type I system, so it was of interest to clarify whether a specific link existed between CARF domain and type III systems and whether or not this linkage depended on the presence of a C-terminal catalytic effector domain in the CARF-domain proteins. To address this question, we compared the co-occurrence of at least one CARF-domain protein containing a (predicted) effector domain with type I, type II, and type III CRISPR-Cas systems (Supplementary File 9). The data presented in Figure 1D clearly demonstrate a strong, specific link between CARF proteins containing a C-terminal catalytic effector domains and type III systems. This association suggests that CARF-domain proteins with this type of architecture play important roles in the majority of type III systems.

## Discussion

Multiple lines of evidence from structural analysis and contextual information from domain architectures and gene neighborhoods suggest that the CARF domains are dedicated ligand-sensors that function primarily in the context of defense against invasive nucleic acids in prokaryotes. Moreover, in the majority of cases (Figures 1A,B) CARF-domains are fused to C-terminal catalytic effector domains, most often nucleases. Thus, it can be predicted that the primary function of CARF-domain proteins is coupling of the sensory stimulus from a ligand to an output in the form of the catalytic activity of the C-terminal effector domains.

The domain architectures of the CARF proteins show certain parallels to those containing the WYL domain: both domains combine with predicted DNA-binding wHTH domains and/or catalytic effector domains. This similarity of domain architectures implies analogous general functions for the CARF and WYL domains which involve sensing soluble ligands in the context of host-virus conflicts. However, unlike the CARF domain, which commonly combines with C-terminal enzymatic effector domains when encoded within CRISPR-Cas loci, the WYL domains appears to be primarily coupled with wHTH domains in the same contexts. Thus, in CRISPR-Cas systems, the WYL domains are predicted to primarily couple ligand-sensing to transcriptional regulation and less often to direct regulation of effectors that target alien nucleic acids. Some families of CARF proteins, such as Csa3 and NE0113, that lack C-terminal effector domains, and the divergent RtcR-like family domains that are linked to the NtrC-like AAA+ domains are predicted, respectively, to regulate transcription directly or via sigma-54. Taken together, the observations presented here raise two key questions: what are the ligands recognized by the CARF domains and what are the targets of their associated effector domains?

With respect to the nature of the CARF domain ligands, recent comparative genomic analysis (Iyer et al., 2013), together with biochemical data (Miller and Warren, 1984; Wiatr and Witmer, 1984; Witmer and Wiatr, 1985; Gommers-Ampt and Borst, 1995), indicate that prokaryotic viruses produce a wide variety of modified nucleotides both *in situ* and as free NTPs as part of their restriction-avoidance and epigenetic regulatory strategies. Many prokaryotic viruses also encode NAD-utilizing enzymes that modify host proteins, in particular RNA polymerase, with ADP-ribosyl moieties (Wilkens et al., 1997; de Souza and Aravind, 2012). Moreover, cyclic 2′–3′ phosphates and their derivatives produced as a result of cleavage of viral mRNA or host tRNA by host RNases during viral infection could also serve as potential ligands (Tanaka et al., 2011). Furthermore, comparative genomic analysis of the counter-phage Ter system has revealed the presence of a cluster of genes that are predicted to encode enzymes involved in the synthesis of a nucleotide-derived metabolite (Anantharaman et al., 2012). Complementary to this plethora of (predicted) ligands, bacteria have evolved several dedicated domains to recognize modified nucleotides in DNA as a part of their bacteriophage restriction strategies (Iyer et al., 2013). Given the prediction that most CARF domains bind negatively charged ligands, such as nucleotides and their derivatives, we hypothesize that at least some of the aforementioned virus-induced metabolites are ligands of the CARF domains. Multiple ligand recognition steps might be critical for the tight regulation of defense systems, such as CRISPR-Cas, whose unchecked activity could have deleterious consequences for the cell (Stern et al., 2010; Makarova et al., 2012, 2013; Dy et al., 2013; Jiang et al., 2013; Koonin and Makarova, 2013; Sorek et al., 2013). Transcription factors containing WYL and CARF domains could act as regulators that tightly control the expression of defense systems unless a specific ligand is present either to relieve the transcriptional block or activate transcription. This is consistent with the recent results showing that a WYL domain protein (sll7009) is a negative regulator of the I-D CRISPR-Cas system in *Synechocystis* sp. (Hein et al., 2013).

We failed to detect CARF or WYL domains in eukaryotes despite extensive sequence searches. The apparent absence of these domains correlates with the conspicuous absence of R-M or CRISPR-Cas systems in eukaryotes. Conceivably, the disruption of operonic organization of co-regulated genes that was apparently associated with eukaryogenesis exacerbated the deleterious effects of these defense systems, leading to their elimination along with the dedicated regulators (Burroughs et al., 2013a; Koonin, 2014). Furthermore, the loss of CARF and WYL-domain proteins, which are predicted sensors of nucleotide derivatives, in eukaryotes is consistent with the limited use of modified nucleotides by eukaryotic viruses (Iyer et al., 2013).

As for the targets of the C-terminal effector domains of CARF proteins, several hints are offered by the parallels with classical Toxin-antitoxin systems and polymorphic toxin systems in which domains of the same families have been identified. In these systems, the RNase domains, such as HEPN, RelE, and PIN, primarily attack host tRNAs or mRNAs and induce dormancy or programmed cell death by inhibiting protein synthesis (Yamaguchi and Inouye, 2011; Zhang et al., 2012; Anantharaman et al., 2013; Makarova et al., 2013). Coupling between such a toxin-like function and interference provided by Cascade-like complexes is most likely ancestral among the type III CRISPR-Cas systems, in parallel with the association of Cas1 protein, a universal component of CRISPR-Cas systems, with toxin-like nucleases Cas2 or Cas4 (Makarova et al., 2012, 2013; Koonin and Makarova, 2013). The fusion of a wHTH domain with many CARF domains suggests that the respective proteins specifically bind DNA. Indeed, REase domains which are present in several CARF proteins typically targeting alien DNA whereas self DNA is targeted only under exceptional circumstances. The REases achieve this selectivity by either targeting DNA with specific modified nucleotides, such as hydroxymethylcytosine (e.g., Mrr, McrA, and McrB systems) (Bickle and Kruger, 1993; Burroughs et al., 2013b), or by targeting unmodified DNA in contrast to the host DNA that is methylated by cognate methylases (Roberts et al., 2007), and probably also by using RNA or DNA guides supplied by Argonaute (PIWI) family proteins (Makarova et al., 2009; Burroughs et al., 2013a,b; Olovnikov et al., 2013).

Thus, we propose that CARF proteins containing C-terminal REase domains function in parallel with the Cascade-like complexes resulting in a double-pronged assault on the invading DNA. In contrast, several bacterial HEPN proteins, such as LsoA and RNase LS, are RNAses that target ribosome-associated mRNAs of infecting bacteriophages, and similar predictions have been made for many other HEPN proteins (Anantharaman et al., 2013). Thus, some of the CARF proteins that contain the HEPN domain and other (predicted) RNAses might act directly on viral RNA to augment the attack on viral DNA or RNA by the type III CRISPR-Cas systems.

The present analysis of the CARF superfamily is expected to provide a new handle on unresolved questions on the regulation and function of CRISPR-Cas systems. Furthermore, these findings could offer leads for biotechnological applications involving ligand-induced action on nucleic acid targets.

## Materials and methods

The Refseq database (February 2013 release) was used to search for CARF domain-containing proteins and analyzed their genomic context in 2262 completely sequenced prokaryotic genomes. The set of 659 representative genomes was selected for quantitative analysis of co-occurrence of CARF-domain containing proteins and CRISPR-Cas systems as follows: for each genus, a species with the largest genome was selected except for the genera *Bacillus* and *Escherichia* for which *Bacillus subtilis* 168 and *Escherichia coli* K12 *substr.* MG1655, the model organisms, were selected for respective genus.

Iterative profile searches with the PSI-BLAST (Altschul et al., 1997) program with cut-off *e*-value of 0.01, composition based-statistics and low complexity filtering turned off were used to retrieve homologous sequences from the Refseq database. In each iteration, all detected sequences were examined for conserved motifs to detect either potential homologs below the cut-off to be included in the profile or potential false positives to be excluded. For borderline cases, additional profile-profile searches were carried out using the HHpred program with default parameters to evaluate the veracity of those matches (Soding et al., 2005). The HHpred program was also used to detect remote homologous families with query sequences selected for each CARF family. Similarity based clustering was performed using the BLASTCLUST program (ftp://ftp.ncbi.nih.gov/blast/documents/blastclust.html) to cluster sequences at different thresholds. Multiple sequence alignments were built using the MUSCLE (Edgar, 2004) program, followed by manual adjustments on the basis of PSI-BLAST and HHpred alignments, secondary structure prediction and structural alignments (if applicable). Protein secondary structure was predicted using the JPred program (Cuff et al., 1998). Transmembrane segments were predicted using the TMHMM version 2 program (Krogh et al., 2001). For each of these programs, unless specifically mentioned, default parameters were used. For each CARF or WYL gene, the gene neighborhood was comprehensively analyzed using an inhouse Perl script. The scrip either the PTT file (downloadable from the NCBI ftp site) or the Genbank file in the case of whole genome shot gun sequences to extract the neighbors of a given query gene. Usually we used a cutoff of 5–10 genes on either side of the query for initial screening. The protein sequences of all neighbors were clustered using the BLASTCLUST program (ftp://ftp.ncbi.nih.gov/blast/documents/blastclust.html) to identify related sequences in gene neighborhoods. Each cluster of homologous proteins were then assigned an annotation based on the domain architecture or conserved shared domain. The Pfam database was used as a guide to make preliminary domain identifications followed by detailed analysis (Finn et al., 2014). This allowed an initial annotation of gene neighborhoods and their grouping based on conservation of neighborhood associations. This was followed by detailed manual analysis of exemplars of each class of neighborhoods. Known *cas* genes were assigned using respective Pfam profiles (Finn et al., 2014) and manual annotation. A complete list of Genbank gene identifiers for CARF proteins investigated in this study is provided in the Supplementary File 1. Structure similarity searches were conducted using the DALIlite program (Holm and Rosenstrom, 2010). The detection of pockets in the structure was performed using the PyMOL Molecular Graphics System, Version 1.5.0.4 Schrödinger, LLC (http://www.pymol.org/) with the Surface→Cavities and Pockets only option. The predicted ligand-binding residues were inferred from the alignment provided in Supplementary File 3.

### Conflict of interest statement

The authors declare that the research was conducted in the absence of any commercial or financial relationships that could be construed as a potential conflict of interest.
