# Supplementary material for: CARF and WYL domains: ligand-binding regulators of prokaryotic defense systems
Source: Front Genet. 2014 Apr 30;5:102. doi: 10.3389/fgene.2014.00102 (PMC4012209; doi:10.3389/fgene.2014.00102)

A. Multiple Alignment of WYL domain.

The domain architectures are shown on the right.The homologs 1sg5A YaeO, a Rho-specific inhibitor of transcription termination, and YolD are also aligned.

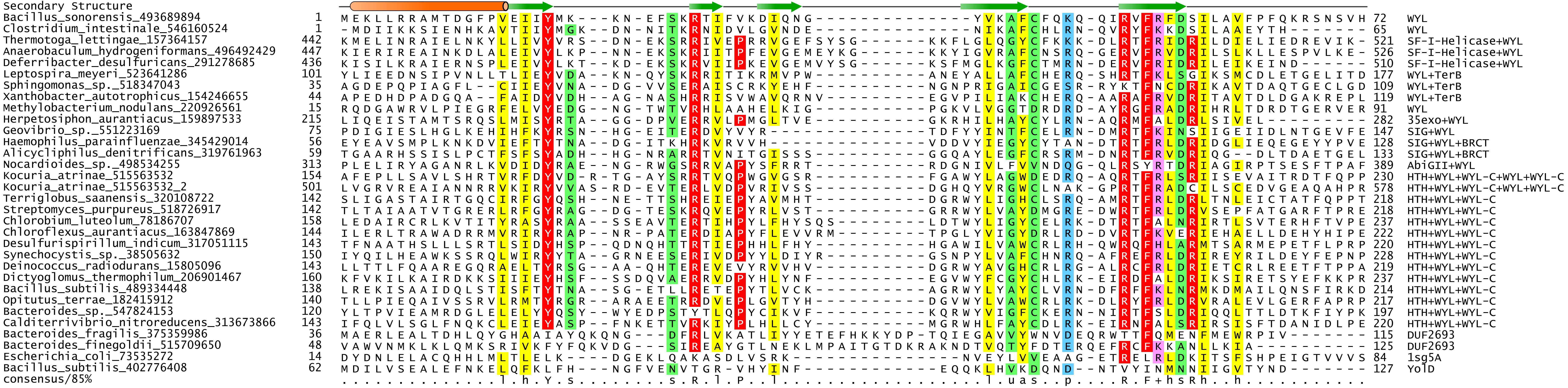

B. Multiple Alignment of the WYL C terminal extension. This domain is primarily found C terimanl to the WYL domains in proteins where the WYL is fused to wHTH.

```
Secondary Structure
Arthrobacter_crystallopoietes_491413391   220  RQDLAGYVQESIVRSPYRYDLV--VRIHAPV--EQVAEKVPPQ--VA--ELE-DDGGSTLLRA-GSDSLDYPLVQLAAMDFAFDILEPPELRTGPWTSARSCVRPDAWSVRP 321
Nocardia_nova_576085320                   246  GGDAANFVRDSL----YSLAPV--HRAEITVF-AGAAEIAATG--IG--EVEAADEHSCRVTT-AADTLPWLLRLIRLGHDFRVDGPPELAAYVGEVGERLQRAAPANRPS 345
Amycolatopsis_methanolica_516609120         1  ---------------------M--VTLHAPL--EEMRPRVG-N--AG--ELTATGPAACRVET-HADTLDWLAFRLLALGCEFEVHEPPELAAHLRALAGRITRATSG---- 77
Terriglobus_saanensis_320108722           219  SFDARRYLAEHMPFVQSDYQID--VWIDMPI--EEANRSFALW--RV--ATE-AHEGGTRLRC-GRDHLEMLAFAAVLLSMRRRIVVHSPPSELRETFRELARLAIQVADGPCAD 320
Deinococcus_radiodurans_15805096          220  DFDALAFLQQELPAA-HPYEIS--VWLAAPP--EHLRGAVSVW--HT--DLR-AEEDGTRMTC-QRENLHSFAAFLLGVGVDFRVDSPPELREVLRTLAGRCQQAGEG---- 316
Streptomyces_avermitilis_29831314          58  GLDPVETLEEHFADG-CKYQVE--VVIDAPA--DTVAEWLPRN--LG--RREPIDAHTTRLVA-TTDEPDWYAEQLTVIKAPFRIVGPPELREAEAVGRRLLGAAGAGRP- 158
Streptomyces_purpureus_518726917          221  KGDAARFLQHSMSRLQPQLDLD--VTFHASE--EFVAARLPAH--LR--PEP-RSATTCRLRVRSSESVEWLAVRLAMVDCPFTVHAPESLITYVSGLADRLAHAVR----- 318
Frankia_sp._493586595                       1  ----------------------MIVDLPA--AFVSQFSRDG--IV--EE--LGPNRCRLVA-GSWSWPSLAATVGRFDADIEVLGPPELADAFAMLARRYARAAAGQRGG 79
Chlorobium_luteolum_78186707              238  SFSIDEYLEKTFDLVHSSEQID--VTIRFTP-FQAQWIRERSWHPTQE-TIE-HKDGSLIMKM-KVGALDAVKRWLMGFGEEAEALEPPQELRILLHDELSRANNLYEDVRAK 343
Chloroflexus_aurantiacus_163847869        223  GFDPYDHLARAWAVM-SEAEVT--IHLRFTP-QVAPRIRENRWHHSQ--QLIDNADGGCDLHL-TVGGIREILSWVLSWGADVEVLAPEELRNEVINHARQMLARYV----- 322
Chlorobium_phaeovibrioides_145219792      123  GFSCDSDLSMSLEQRQGVPRAH--VVITFSS-HHAQWIRTGMWHSAQ--TFQEHGDGSVTLSM-DVGSLEGVKRWVMRFGAGVRVISPEELRNMIKDEVVRMGMVYLDMQDS 228
Dictyoglomus_thermophilum_206901467       238  DFDVYEYIKSAFRI--NKGDVE-KIRIKFDP-YQARWIKERIWHETQ--SIEELPDGSIIYEI--VANRDEIKRWVMGYGKHAEVLEPESLRNEIREEIKKLGEIYKVL--- 338
Frankia_sp._517469060                     258  RYEAEVYRSSATVRL-SPAGLRL-VPFRLAP-AVARSVQRT-----AG--EP--DADGWVRARL-PIESVRHARGDLLVLGADVEVLGPPELRAAMTEAAADLTALYDSPPG- 356
Synechocystis_sp._38505632                229  NFDWKKYSKNAFQIEKGNCLHE--VVIDFTC-KVAPYIREWKWHHSQ--VIDEHHDGSLTLHL-EVGGLQEVKRWVLGYGKDALVLKSPPELVKLLQEETEVIARQNEDGDFE 334
Sphingomonas_sp._497508927                 31  DWDIDAWLADSFGVW-RDEKQD--VVLRIHA-SAASRAREWRFHRNQ--QIEQLENGDLLIRF-TSGGMRELAEHLFTWGNELIIEQPVALIEMMR---QRIMAALTMLPPE 132
Nocardia_brasiliensis_407644347             1  ----------------------MTRPCRCAHEAAR-----------QG--VLESIDESRCLLTT-FPDTPQYLAYNIALLPMPYTLLEPAEVAQHLREMAERAATATAHLTEP 77
Streptomyces_purpureus_518731544            1  -------MPTRYRAV-----------ATVPAPADHIRARTRARTRGLAT--RVRPVDERTCRVDA-SDDSLPRIARTLAWLDTDYTLDADPEVLDHLRATAHRTLRATG---- 88
Treponema_phagedenis_488784522              4  DFNPEKYIDKNIGVWLTKREPF-TVRLLFSN-EVGVFAEERMWSKEQ--TVTVHDDKTVEVSF-KTTQLEEIKRFVLGQGATVQVLEPQELIDEVRAELEKMRTLWKCQYQF 110
delta_proteobacterium_494502338             7  AAAIISISSQSYGIFRGGRQLR-QVTLQFDP-HMARLVSGMFWHPEQ--QGESTPDGVLRLTI-PVADFTEITMRILSFGRHVEVLAPEELRRRVAEEVGEMAEMGGKTP-- 111
Opitutus_terrae_182415912                 218  DFSLRRFLEQNMD---DRERVA--AKIWFAE-DALDRVRRESYNGVV--ALR-PEGAGAEVEL-KTFSLEWLARWILSFGAEAEARSPAKLRALVRQEAEAVAQRYGDA--- 316
Roseiflexus_sp._148658418                   1  ---------------------M-LRFSP-HVARRVQETRWHRSE--RTEPQPDGSLVWRA-QIAEPKEMLPWIRGWGADVEVLGPEGLRGTLAEVRKMARVYGVSEMK 83
Salinispora_tropica_145595271             123  GTDAGTCCATHTARMRSART----VSIGFGR-----------CG--PLEPDGDRCVLVGS-TRNPTMYAQEWLAAAPFDFSVEEGAELRAAVQASRLASSGHFHLRGPK 213
Sorangium_cellulosum_162450719            396  DFSIDDYFQGQFGIWRGGEPRR--VVIDFDE-RVTEFVRTRKVHPSQ--EITPLSGGGVRLTM-LIGDMTELATWVLGFGETARVVEPPDLVDRVKRELAGALARYEEPPSP 501
Mariprofundus_ferrooxydans_517105065        5  EELNEHLGTSYGIFSGKADKL--ALLRFSP-EARWVSAETWHPEQ--KGTWQEDGGYELRL-PYFNPTELILDICRYGPDVQVIEPDELREAVTERLRRAADQYN----- 105
Clostridium_asparagiforme_494988983       113  PRDYPPYDPSPEKVFPAALQ----ATALFEP-SMKWRLMEE--YGPE--SFEETEDGRLLFTC-GFTDVESLFGWLLSFGPTVELLEPEKLRPRFRELVSDIIKKYNGDV--- 212
Bacillus_coagulans_516121541               12  GFRLADHRPA-------DDRKLT--VRVRVDA-AVADQVRETGSFYLE--SFEPGEDGSWWLATFRVRRIEELLRWTLGWGANVEVQVLEPESMRERLREEIGKMRKNY---- 106
delta_proteobacterium_494505238             7  AAAIISSSQSYGIFRGGRQLR-QVTLQFDP-HMARLVSGMFWHPEQ--QGESTPDGVLRLTI-PVADFTEITMRILSFGRHVEVLAPEELRRRVAEEVKRPQPSVEMKVMI 113
Eubacterium_siraeum_547864879              62  TYKESDYLDD-FGMKKNGEWYE--IVMLLHG-RYAMLARERIYGRDQ--TVTPIDEDTTELRC-TMQNEENIMCFVMGFGSHCEVVSPQWLKDRVKDEAEKILSQSIR---- 162
delta_proteobacterium_494502342            54  RYDVDRHIRQSYGIFKGGRQLR-QVTLQFDP-HMARLVSGMFWHPEQ--QGESTPDGGLRLTI-PVADFTEITMRILSFGRHVEVLAPEELRRRVAEEVGEMGKKTRPARER 160
Eubacterium_sp._547751772                  19  NFDIADYSKKTFGMF-GGREQT--ISVQCKNSFVGVFIDRFGDSVSI--RPNYENPDYCICRM-TVNVSPQFYGWIFALGAEVTIVSPQSAIDEFREMAKTIFENYNRN--- 121
Rhodococcus_rhodochrous_515066663           1  ---------------------M-TITVRIAH-DVSDKARESRFYYID--AIQDHPDGLLV-TL-RVRQPEEVLPWVLGWGSKAVVVEPESFRLHVRKEIENMLKHY----- 79
Desulfurispirillum_indicum_317051115      220  -PAEQWQSLVNHAYGLFQGKLTTAVRLRFSP-ETSRWIREQQWHPAQ--TMREETCGAVELSF-PVADLREIKMRVLSYGAEVEVLEPVELRDMILAEAKKMVGESE---- 321
Streptomyces_sviceus_494627954              1  ---------------------MRVAPGV-RLPRAHECV----------DSDGWTLARV-PIESLGHAQGEFLRLGADIEVLEPAELRARMARTISELAERYGNSASP 75
Paenibacillus_sp._496516636                 1  -------MTDTDPLIDTGQTVRVRARFYNPEGSVPNFVKERVLLQGQWGQIVFEDENSFIYEI-TVNGTTEIKPWLRSFGSCEVIEPASLRREMIAEWKEILSYYESV--- 101
Firmicutes_bacterium_556500774             63  -GFDPAVYVHQVFGMF--GSDIRRITLLCEN-STMRSVVDR-FGEEV--KTEIVDGEHFQATV-DVAPSPPFFAWVFTFGGKIRITEPEEIAVKMREMAKWLQ-------- 157
Lachnospiraceae_bacterium_547738578       201  KKLDMADYAKKSFGMFRGNERT--VRLLVKN-NLAGVIIDR-FGKDV--RMIPADEQHFTVNV-DIHVSKQFFGWVFSLGGEGIKIIGPGEVEEQMKEEARRLLEQYENKGDA 305
Streptomyces_sp._518970822                 75  AERFARSILRAEVVLRLSATGVRNLPYAVDP-VAARDALAG--------VPQPDATGWATVTL-RVESEEVAHTQLAALGPEAEVLFPATLRERFAADARRLTELYGRREG- 175
Streptomyces_sp._496483969                265  SEAFARGMLRTTVTLRLTEDGLRRLPAVLDP-AAAGALPVC--------APPRDAAGRVTLDV-PVESEDVAFGQLTALGADAEVLAPASLRARFRAYARSLAARYDAEP-- 364
Conexibacter_woesei_284047221             597  SRDVEGWPRT-----GEVEASRRARVWISP-ERARWAREE----RS--VVEELADGAVVVEL-SFAGLDWLVREVLKEAGDAAVLEPDDARTAVRVAANRLVRV------- 687
Alistipes_shahii_479185744                197  ELDPAGYLAGAFGVAVYDDICPCTIRVRVAG-DGVKYLRTLPLHASQ--QEVETHDDYAIFEF-HVAPTPEFYAQMLNCHCNFEVLLPDNVREEMRRIIGDLGALYDHNVQK 304
Bacillus_subtilis_489334448               216  QLDTLPWDRNWY----EKDRLT-ELVILVQQ-PARQRIRE--WFGSE--VLHCDEDENCRAVI-SLPEDQWLIGFLLQFGKDIEVLEPLHIRDKVKDTIHQMQKVYET---- 312
Nocardiopsis_gilva_516212687              423  RARRRDLSGGVLQL--SDDAR--VLLDLES-SARWVTEDYLCS--VVRELPDGRLRATL-RTPAPAWACRLALRLGPRAVVAPADLAERVREDARRALSAYAGNG--- 521
Actinopolymorpha_alba_522067876            16  DFDLTQYWAQAQRQFRATLPSY-PILLRVRD-HAVRRFTA-----TA--PARPDIEGWSLVEA-DLENADEACAAVLAQGGDAVVIAPADLAERVREDARRALSAYAGNG- 112
Streptomyces_sp._518594315                 62  TDFARSLLRTEVTVRVSESGAR-QLAYAGDR-AAAAEALAQ--------APPAGPDGWRTVVL-PVESLDVAYSQLLSLGPELEVMAPEELRGRFASAAERLIRELYR---- 157
Arthrobacter_crystallopoietes_491413391   220  RQDLAGYVQESIVRSPYRYDLV--VRIHAPVEQVAEKVPPQ----VA--ELE-DDGGSTLLRA-GSDSLDYPLVQLAAMDFAFDILEPPELRTGPWTSARSCVRPDAWSVRP 321
consensus/90%                                  ........................h.h.h...............................h............h...l..hs..h.l..P.pl...h......h......
```

C. Cartoon representation of 1sg5A (YaeO, a Rho-specific inhibitor of transcription termination), a sm-like SH3 β-barrel fold homologous to the predicted WYL structure. The figure shows the surface in grey, the binding surface in pink and the positions corresponding to the conserved residues in WYL, that are predicted to be involved in binding, as spheres.

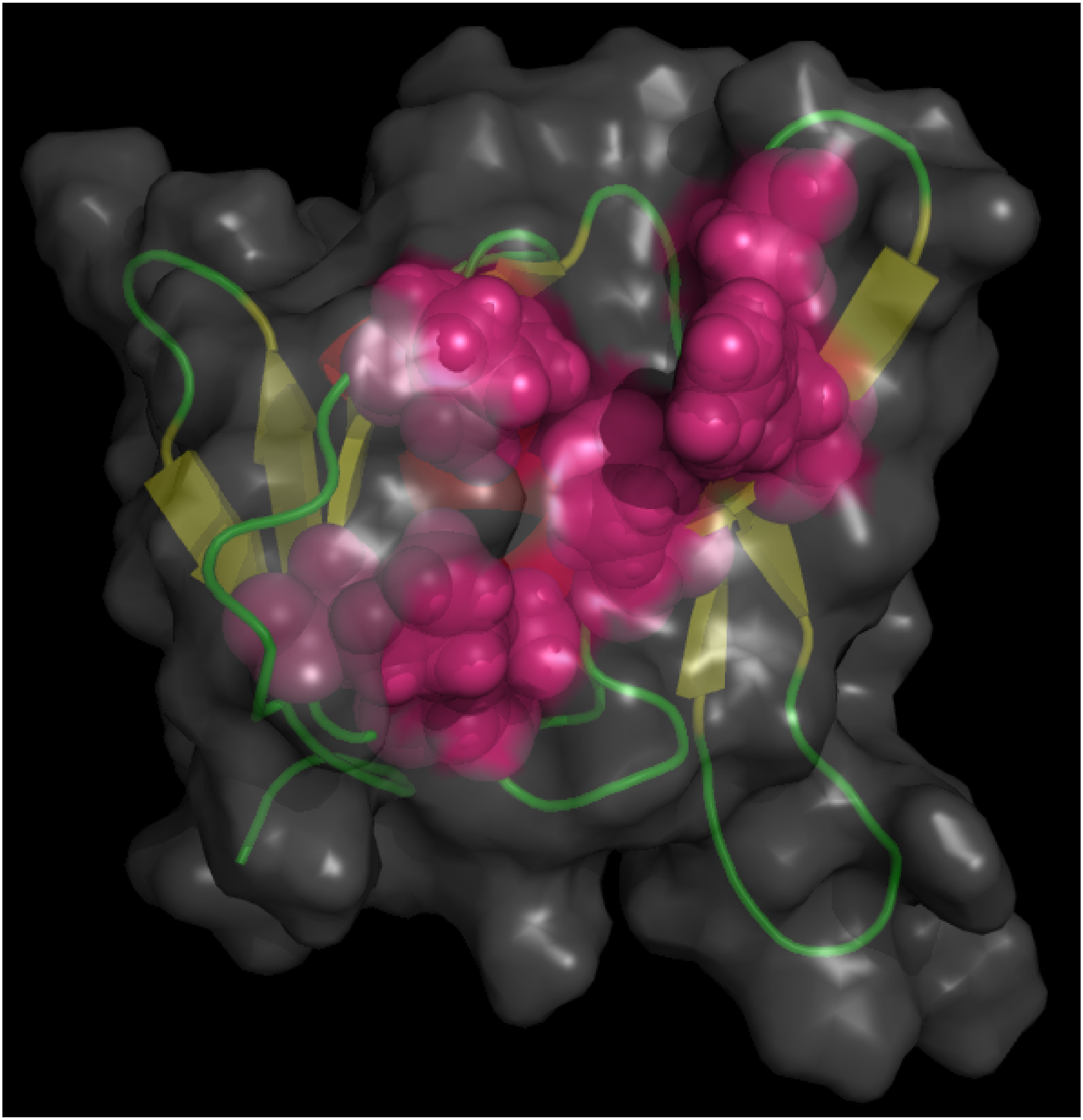

Supplement: Supplementary file 5 [file DataSheet5.PDF]
